# Letter to a Young Physician

**Published:** 1853-11

**Authors:** 


### IOWA MEDICAL JOURNAL.

VOL. I. KEOKUK, IOWA, NOVEMBER, 1853. NO. 4.

### ORIGINAL COMMUNICATIONS.

### *Letter to a Young Physician:*

JUDGMENT AN ELEMENT OF PROFESSIONAL CHARACTER.

I am anxious to impress upon your mind the fact, that no one requisite of a physician's character is of so much importance as a *sound judgment*. You know very well that the practice of medicine calls for a full exercise of the reasoning faculties. You have, I doubt not, been taught that every case of disease is, of itself, a demand upon the energies of the intellectual faculties. To calculate with accuracy and success, the various elements of disease, to weigh carefully, their discordant forces, to sum up the conflicting testimony, and decide upon a satisfactory and successful course of treatment, this is by no means an easy task. The reason why I am desirous to impress upon you a sense of the importance of a sound judgment is, that although the basis of it must of course be an inborn faculty, yet it may be much improved and strengthened by careful culture. But fix in the mind an earnest desire to strengthen this invaluable faculty, and let this desire be the stimulus of a daily and hourly effort to toughen the judgment into a masterly vigor, and you will not be disappointed. Habits of cautious and discriminating thought will improve it; and so will the most useful custom of asking yourself, in the leisure of every day, the causes and effects of the myriad phenomena around you. The possession of this faculty was one trait which distinguished Newton and Franklin from common mortals, and the rigid cultivation of it, will go far towards making a medical philosopher.

Few things, again, so strengthen the judgment as the invigorating process of questioning, by yourself, the propositions of others, and admitting nothing to your assent till it has passed the ordeal of a searching criticism. Go carefully over the great models of logic and mental acuteness, until the torturing discipline of the mind shall be a thing of ease and pleasure. It is said of a celebrated statesman that he carefully went over Euclid's Geometry every year, and I am sure you would not doubt but he was a better defender of the nation's rights, for his study of angles and curves.

A physician should train himself to the custom of frequently going over, in his mind, the process of reasoning, on which he bases his treatment of disease, and asking himself if he has, by any chance, been misled; if the premises could warrant any other conclusion; if sources of error may not have crept in, and warped his judgment. I beg to urge this upon you, as a most useful habit; you will often find that the calm and thoughtful meditation of the evening, for instance, will correct the errors of the morning's agitation, or may perhaps throw new and valuable light upon some important topic, which you had previously considered fully matured. By all the means within your reach, learn faithfully to trust to the guidance of your own intellect. It is a source of most lamentable error, this relying upon the direction of others—moving upon authority; and few customs are so habitual and so pernicious to medical men.

You should trust no man's judgment, so much as your own; that is, if the facts are as obvious to you, as to others. Not that you are to consider your judgment the best in the world, but regard it, as it really is, or can be, the best *for you.* Cultivate your own judgment, and it will, by and by, prove itself worthy of culture. Nourish and sustain it, in youth, and in old age it will nourish and sustain you. Strength of decision and soundness of judgment are the only means of real independence. Confide in your own mental strength; it may possibly, at first, lead you astray a few times, but invigorate it by manly encounter and a trusty reliance, and it will soon repay your labor, and reward the amplest confidence. Fear not the first trivial mishaps to which the rough discipline may subject you; better, by far, to stumble upon your own hobby, than to break your neck upon your neighbor's.

One element of a good judgment is penetration. Two or three times in a century some great mind is born, with an apparently intuitive faculty of distinguishing almost at a glance the true from the false. But ordinary mortals, like you and me, must learn, by the slow process of laborious training, to exercise a similar subtelty. As the physical senses may be educated to a keener perception of the outward world, so may the mind's eye be trained to a nicer discrimination of the objects of mental vision.

In order, however, that the exercise of judgment may be the more accurate and unerring, the mind must be trained to act, amid urgent necessities, with directness and composure: so that self-possession is another element of a good judgment. Haste makes waste, the world over. In every circumstance of life, we bestow large confidence upon that man whose well trained mind is undisturbed by the wild tornado that frenzies common spirits, but whose serene thought rather gathers strength from the commotion going on around it. We envy the calm majesty of a great and quiet soul; we know that true and enduring purposes rarely come from the hasty impulse of passion, and that no reliable principles of action are likely to be generated during the hesitating timidity of a trembling spirit. The want of entire self-possession is the besetting danger of many physicians. The sufferings of the patient—the urgent entreaties of his friends—and the physician's own anxiety, all tend to divert his mind from the calm course of thoughtful study; and he is fortunate indeed, if in his earlier years, he be able, under such moving influences, to preserve the unbiased integrity of his judgment. Instances are of every day note, where physicians of eminent ability, dare not attend upon the sick members of their own families—so strong is the fear, lest the predominance of the affections and sentiments, should swerve the judgment from its proper course of action.

The perplexities of an obscure and difficult case, you will find to be amply sufficient of themselves, without distracting yourself regarding the high station, or peculiar urgencies of the present case. Most persons think to augment the chance of the patient's recovery, by constantly accumulating, upon the physician's mind a deep sense of the immense responsibility, in this particular case. Napoleon was wiser than this. His deportment indicated the shrewd severity of his common sense. During the sickness of the Empress, he perceived that the physician was embarrassed by the rank of the royal patient, and that he feared lest his timidity might prove fatal to her. Did he take the physician aside, and appeal to him upon the intense responsibilities of the case—did he tell him that the hopes of millions rested upon the course pursued? His order to the physician was "go on and attend to her, as to any one else—she is but a woman—do by her as you would by the wife of a fishmonger." The medical attendant regained his courage, and the royal patient her health.

A good judgment, then, supported by penetration and self possession, forms an indispensable element of the character of a good physician. But this can hardly be fitly exercised, without contsant attention to another important point, accurate observation. It is the testimony of one of the most eminent of physicians, that so far as he had witnessed, professional superiority depends, not so much upon mere genius, or any abstract mental quality, as upon long continued habits of severe and rigid observation of disease. Some persons seem to infer, as a matter of course, that a large practice, or, in other words, much experience in the treatment of disease. necessarily involves profound wisdom—an inference which is a profound blunder. Forgetting that many practitioners, having eyes, see not, they take it for granted, in their estimate of a physician, that the amount of his wisdom is in direct ratio to the greyness of his hair. Two persons shall visit Europe for a summer. One will return, freighted with entertaining and useful information, of all kinds, regarding every country he has visited, and the other shall come home, with little else to inform you of, than the modes of travelling, and the bills of fare at the principal hotels. As various as this, are the observations of different physicians, in the sick chamber.

A good judgment can neither be formed or strengthened, without a rigid study of the causes, forms, relations and effects of every phenomenon which presents itself to you. By such a study, and careful observation of the various processes of disease, you will advance every day in skill, and true wisdom; for such study is the original and only source of all knowledge. Without such a course, the practice of one science degenerates into a mere mechanical trade.

Now, I know some of the points I have hinted at, appear to you quite difficult. It seems a labor to note in one's mind so large a store of daily facts, and to combine, harmonize and select, so that you may advance daily in information and ability. I have to inform you that it is easier by far, to creep stupidly on, with half closed eyes, and brain half asleep. But, if it seems too much a task—if even after having entered upon your profession, you seem to half-hesitate, at the life long labor—now in the best stopping place. Better by far, not spend any longer time in such a pursuit, if you cannot give to it your undivided energies. Turn to-day from your present employment, unless you are resolved to satisfy a zeal to excel, or at least to meet the demands of an enlightened self respect. J. E. S.

